# Serratia marcescens Tricuspid Valve Vegetation and Successful Use of the AngioVac® System

**DOI:** 10.7759/cureus.10010

**Published:** 2020-08-25

**Authors:** Sean M Winkle, Salem Gaballa, Areeka Memon, Jeremy B Miller, Ryan Curfiss

**Affiliations:** 1 Internal Medicine, LewisGale Medical Center, Salem, USA; 2 Osteopathic Medicine, Edward Via College of Osteopathic Medicine, Blacksburg, USA

**Keywords:** serratia marcescens, tricuspid valve endocarditis, angiovac system, cavitary lung lesions

## Abstract

*Serratia marcescens* bacteremia is common in patient populations with a history of intravenous drug use (IVDU), but it rarely causes infective endocarditis. We are reporting a 27-year-old female with a medical history significant for IVDU and hepatitis C virus infection who presented to the emergency department complaining of fever and shortness of breath. Computed tomography of the chest with intravenous (IV) contrast revealed extensive bilateral pulmonary infiltrates with multiple cavitary lesions. The patient was treated with IV vancomycin and piperacillin/tazobactam. Blood culture grows methicillin-sensitive *Staphylococcus aureus* (MSSA) and *S. marcescens*, both sensitive to cefepime/meropenem. Transesophageal echocardiogram revealed 3.4 x 2 cm tricuspid valve vegetation. Cardiothoracic surgery was consulted, who recommended transcatheter aspiration with the AngioVac® system (AngioDynamics Inc., Latham, NY). Post-procedure transesophageal echocardiogram revealed a significant reduction of vegetation size. Vegetation tissue culture grew MSSA and *S. marcescens*. The repeated blood culture revealed no growth, and the patient significantly improved clinically. She completed a six-week course of IV meropenem as an inpatient until she was discharged home.

## Introduction

Infective endocarditis (IE) is an infection of the inner lining of the heart and generally involves the heart valves but also may occur at septal defect sites, on chorda tendinea, and on the mural endocardium. IE can occur on both native or prosthetic valves. The incidence on native valves is 1.7-6.2 cases per 100,000 person-years and is worsened by risk factors such as intravenous drug use (IVDU), which has an incidence of 150 to 2000 per 100,000 person-years [1]. Other risk factors for IE include male gender, bacteremia, congenital heart disease, disruption of the gastrointestinal tract, poor dental hygiene, diabetes mellitus, human immunodeficiency virus infection, indwelling central lines, and cardiac implantable electronic devices [2]. *Serratia marcescens* IE is an extremely rare infection with no definitive treatment guidelines. In the era of opioid epidemics and increased risk of blood-borne infections, it is essential to recognize *S. marcesens* as a possible cause of IE and initiate the appropriate antibiotics promptly [3].

## Case presentation

A 27-year-old female with a past medical history of IVDU and untreated hepatitis C viral infection presented with subjective fever and chills for one week. Additionally, she had complaints of generalized weakness, fatigue, and nausea. She was treated for sepsis at a neighboring hospital two weeks prior with broad-spectrum antibiotics but did not complete treatment, leaving against medical advice due to inadequate pain control.

On presentation the patient was febrile, tachycardic, and tachypneic, requiring 3 L oxygen (O_2_) via nasal canula. On examination she appeared anorexic, pale, and dehydrated. Cardiac auscultation revealed a 3/6 holosystolic murmur loudest at the left lower sternal border. Bilateral fine crackles were appreciated on auscultation of the lower lung fields. Laboratory data showed as in Table 1: white cell count 16.8 x 10^9^/L with 81% neutrophils, hemoglobin 6.0 g/dL, hematocrit 19.2%, platelets 47 x 10^9^/L, prothrombin time 13.2 sec, fibrinogen 498 mg/dL, D-dimer of 12.9 mg/L, and lactic acid 6.0 mmol/L.

**Table 1:** Laboratory results on admission

Tests	Result	Reference Range
Hemoglobin	6.0 g/dL	14-16 g/dL
Hematocrit	19.2 %	40-52 %
White cell count	16.8 x 10^9^/L	4-10 x 10^9^/L
Platelet count	47 x 10^9^/L	150-400 x 10^9^/L
Sodium	137 mEq/L	135-145 mEq/L
Potassium	3.3 mEq/L	3.5-5.2 mEq/L
Chloride	108 mEq/L	96-106 mEq/L
CO_2_	24 mEq/L	23-29 mEq/L
Blood urea nitrogen	8 mg/dL	6-20 mg/dL
Creatinine	0.6 mg/dL	0.8-1.2 mg/dL
Albumin	1.3 g/dL	3.4 to 5.4 g/dL
Erythrocyte sedimentation rate	52 mm/hr	0-26 mm/hr
C-reactive protein	7 mg/L	0- 10 mg/L
Troponin	<0.015 ng/mL	0-0.045 ng/mL
Lactate	6 mmol/L	0.4-2.0 mmol/L
Total Bilirubin	1.4 mg/dL	0.2-1.0 mg/dL
Direct Bilirubin	0.4 mg/dL	0.0-0.2 mg/dL
Lactate dehydrogenase	249 u/L	84-246 u/L
Prothrombin time	13.2 sec	9.3- 11.5 sec
Activated partial thromboplastin time	29.1 sec	23- 34 sec
International normalized ratio (INR)	1.29	0-1.1
Fibrinogen	498 mg/dL	183-475 mg/dL
D-Dimer, quantitative	12.9 mg/L	0.00-0.59 mg/L

**Figure 1:**
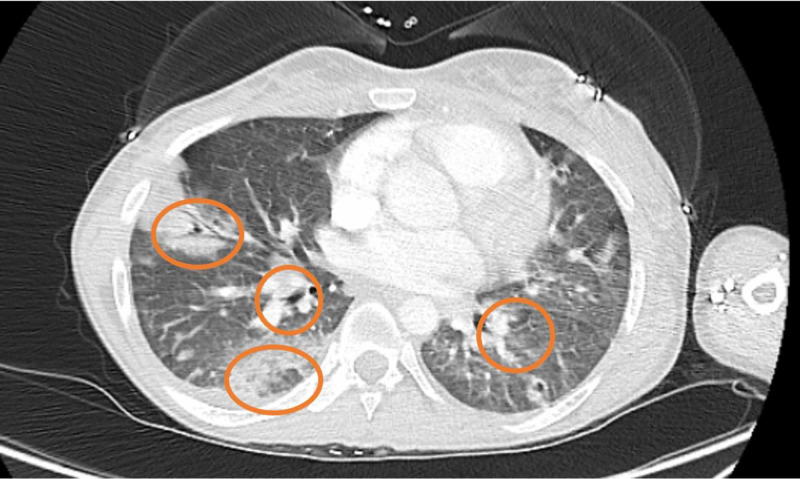
CT scan of chest with IV contrast. Axial view of chest CT (lung window) revealing multiple bilateral cavitary lesions due to septic emboli likely from tricuspid valve vegetations.

**Figure 2:**
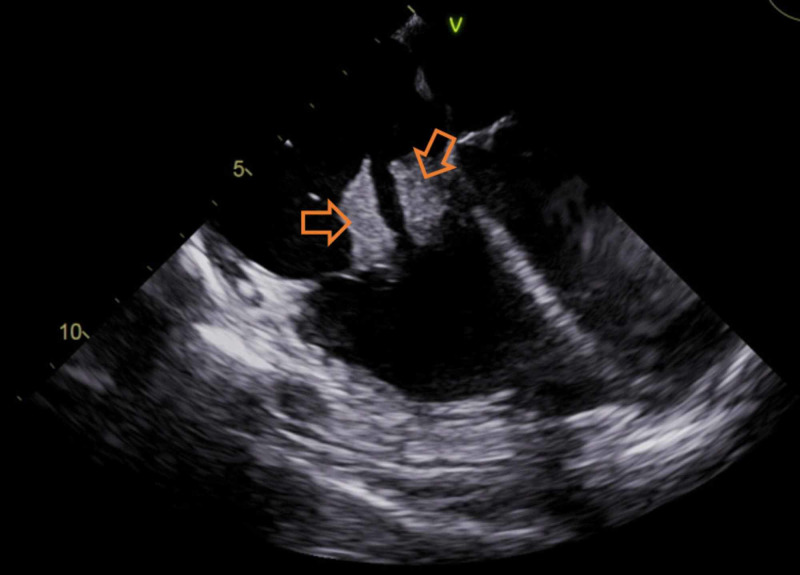
Preprocedural transesophageal echocardiogram (TEE). Multiple large vegatations located on anterior and posterior leaflets of the tricuspid valve prior to percutaneous aspiration procedure.

**Figure 3:**
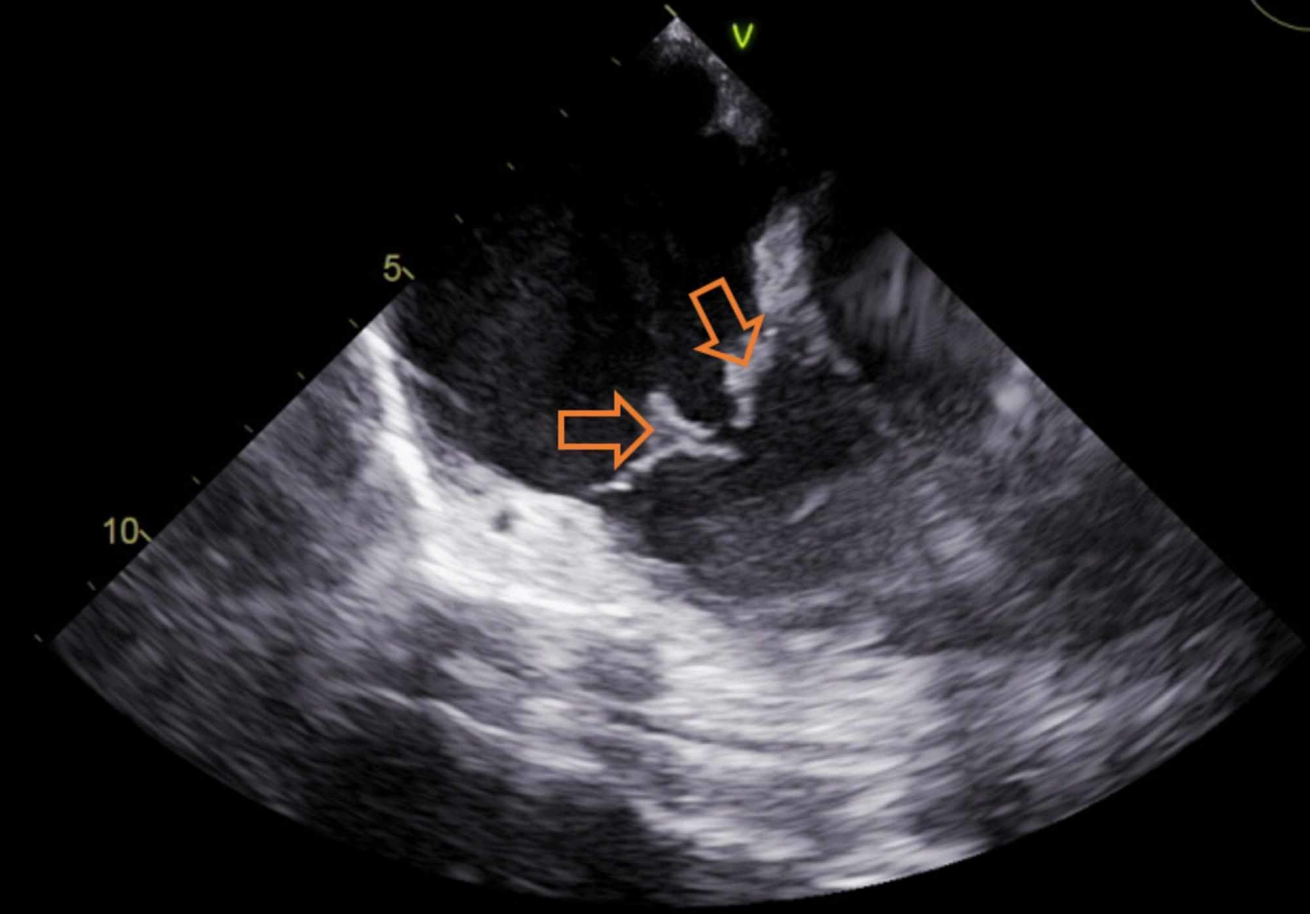
Postprocedure transesophageal echocardiogram (TEE) TEE following percutaneous aspiration of tricuspid vegetations showing significant reduction in bulk of both masses.

**Figure 4:**
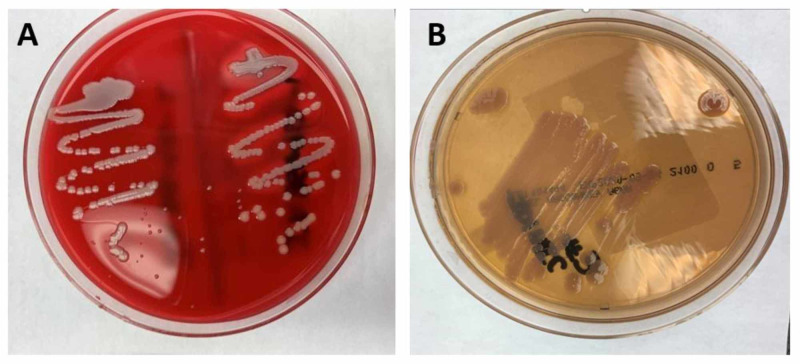
Tricuspid vegetation culture plates. A) Blood agar plate growing methicillin-susceptible *Staphylococcus aureus* (MSSA), B) Modified MacConkey agar growing *S. marcescens.*

**Figure 5:**
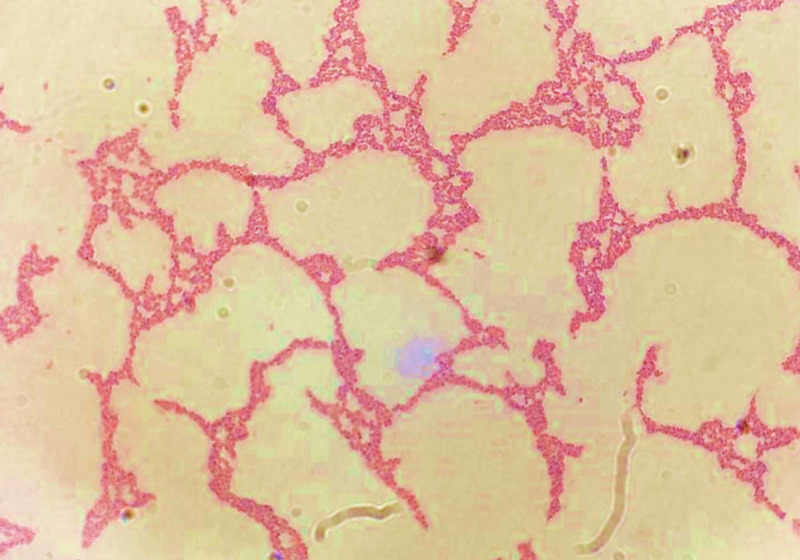
Gram stain of tricuspid valve vegetation. Magnification (400x), showing colonies of gram negative baccili *S. marcescens.*

Urine drug screen was positive for opiates, methadone, and marijuana. Computed tomography (CT) of the chest with contrast revealed extensive pulmonary infiltrates with multiple cavitary lesions, likely due to septic emboli (Figure 1). Broad-spectrum intravenous (IV) antibiotics (vancomycin and pipercillin/tazobactam) were started. She was admitted to the intensive care unit for severe sepsis. A transesophageal echocardiogram (TEE) revealed multiple vegetations on the atrial aspect of the tricuspid valve (Figure 2), the largest measuring 3.4 x 2.0 cm on the posterior leaflet and 3.2 x 1.1 cm on the anterior leaflet. Blood cultures taken were positive for *S. marcescens* and methicillin-susceptible *Staphylococcus aureus* (MSSA). Antibiotics were tailored to microbial susceptibilities. Percutaneous aspiration with an AngioVac® system (AngioDynamics Inc., Latham, NY) was performed for resection of the tricuspid vegetations. The post-procedural TEE showed significant reduction of vegetation size on the tricuspid valve (Figure 3). Cultures of the vegetations grew *S. marcescens* and MSSA (Figure 4 and Figure 5).

Following the tricuspid vegetation debulking procedure and tailored antibiotic therapy, the patient continued to improve significantly. Supplemental oxygen was no longer required, tachypnea and tachycardia resolved, leukocytosis resolved, and coagulation aberrancies normalized. The patient required an extended six-week course of IV meropenem (per susceptibilities) prior to discharge with follow up for continued monitoring of sequelae due to septic emboli.

## Discussion

The majority of IE is left-sided, however 5-10% of IE is right-sided and this is usually seen with IVDU or patients with IV lines and wires placed for medical reasons. Right-sided IE occurs when there is diluent-related damage with thrombus, drug-induced pulmonary hypertension with secondary valve damage, IVDU-related endothelial problems, or IVDU-related immunologic abnormalities [4]. For an infection to be sustained there must be irregularities of the endocardium to allow for bacterial adherence, adhesion must occur, and there should be survival of the adhered bacteria. The endocardium can become more habitable by bacteria through trauma to the tissue leading to deposition of platelets and fibrin. This surface, as well as various bacterial virulence factors such as dextran, promote the adherence of the pathogen. Lastly, bacterial survival is promoted through resistance to phagocytosis by white blood cells [5]. 

The presentation of IE differs based on whether it is right or left-sided. In left-sided IE patients have symptoms such as sustained bacteremia, peripheral emboli, and vascular immunological phenomena. Right-sided IE can present with septic pulmonary emboli. Additionally, chronic IE generally presents with an increased number of symptoms, while acute IE can present with less defining symptoms. The diagnosis of IE may be made by using the modified Duke criteria. This includes a list of major and minor criteria, that when fulfilled, is likely a case of IE. The major modified Duke criteria are blood culture positive for IE and evidence of endocardial involvement. The minor criteria include: predisposing heart condition or IVDU; fever of 38 degrees Celsius; vascular phenomena including major arterial emboli, septic pulmonary infarcts, mycotic aneurysm, intracranial hemorrhage, conjunctival hemorrhages, and Janeway lesions; immunological phenomena such as Osler nodes, glomerulonephritis, Roth spots, and rheumatoid factor; and microbiological evidence. The criteria are fulfilled when one major and one minor or three minor criteria are met [6].

IE can vary in terms of presentation but also has a variety of organisms implicated in the illness, the most common infecting organism being *S. aureus* [3]. Other causes include streptococci, coagulase-negative staphylococci, gram-negative organisms, and fungi [7]. The risk of each of these can vary based on many individual patient characteristics, but the rarest forms of IE include those that are fungal or non-HACEK (*Haemophilus* species, *Aggregatibacter* species, *Cardiobacterium hominis*, *Eikenella corrodens*, and *Kingella* species) gram-negative aerobic bacilli. One rare non-HACEK gram-negative organism implicated in IE is *S. marcescens*.

*S. marcescens* is a gram-negative, aerobic, motile bacillus that produces a red pigment. It was first discovered in 1819, and the knowledge regarding the organisms’ infective capabilities has increased since then [8]. *S. marcescens* is present throughout the environment and can also be found in the human intestinal flora. It has been found in soil, plants, animals, and water sources [9]. The organism is frequently a hospital-acquired bacterium and is known to cause infections such as catheter-associated urinary tract infections, pneumonia, meningitis, and wound infections [8-9]. The first case of *S. marcescens* IE was reported in 1951 and had an association with IVDU [8,10]. Virulence factors of this organism include lipopolysaccharide, pore-forming hemolysin, mannose-resistant pilli, mannose-sensitive pili, chitinase, lipase, chloroperoxidase, and biofilm production [8-10]. *Serratia*, along with some other *Eneterobacteriacae*, have an AmpC Beta-lactamase that is able to hydrolyze penicillin as well as first, second, and third-generation cephalosporins. This enzyme is usually induced when a patient is given beta-lactam antibiotics or if they have a certain mutation that can lead to antibiotic resistance. Therefore, while the antibiotic selection is difficult with *Serratia *infections, susceptibility testing can be utilized to guide treatment [8].

The diagnosis of IE begins with a transthoracic echocardiogram (TTE). Indications of IE on TTE include an oscillating intracardiac mass, annular abscess, prosthetic valve partial dehiscence, and new valvular regurgitation. If the initial TTE is negative despite clinical suspicion of IE, TEE can be done [6].

Treatment for IE includes antibiotics, surgery, or the use of a transcatheter aspiration system (AngioVac® system). Anticoagulation therapy during an active infection is controversial because it could facilitate future embolization and lead to intracerebral hemorrhage. Therefore, anticoagulation is only started for indications other than septic emboli or if a prosthetic valve is involved [1]. Antibiotics are the first-line treatment and should be continued in an individual for a determined course length once there are negative blood cultures. In patients with an active infection, blood cultures should be obtained every 24-48 hours to assess infection status [6]. Surgery is indicated for IE with heart failure, uncontrolled infection, and to prevent embolization. Emergent surgery is recommended for heart failure with pulmonary edema and urgent surgery is recommended for heart failure with poor hemodynamic tolerance [11]. Uncontrolled infections refer to IE with complications such as an abscess, fistula, pseudoaneurysms, or positive blood culture 7-10 days following therapy or resistant organisms. Additionally, surgery done to prevent embolization is based on the size of the vegetation. Surgery is recommended for vegetations greater than 10 mm if the patient has had previous embolization and for vegetations greater than 15 mm for isolated vegetations [12]. Transcatheter aspiration may be considered if medical therapy is failing or if the patient is a poor surgical candidate [13]. The AngioVac® system is a vacuum-based percutaneous aspiration device that is minimally invasive and has been approved for removal of materials from the intravascular system since 2014. The procedure can also be done to reduce complications of surgery, increase the efficacy of antibiotics, and can be used as a bridge to surgery or as an alternative in patients with high perioperative risk [14-15]. Complications of the AngioVac® procedure include vascular or myocardial injury, pericardial tamponade, tricuspid valve injury, or pulmonary embolism [16]. Following treatment of IE, an additional TTE or TEE should be done to establish a new baseline of heart function. Post-treatment, patients are most likely to have a relapse of infection within two months. Cases involving *S. aureus*, *Enterobacteriaceae*, and fungi generally relapse during the primary course of therapy [1].

## Conclusions

*S. marcescens* IE is an extremely rare infection with no definitive treatment guidelines. The AngioVac® system is a promising noninvasive treatment for right-sided IE. The AngioVac® system has shown its efficacy in reducing the intracardiac vegetation size and the associated bacterial load, therefore increasing the efficacy of antibiotics in the clearing of the bloodstream infection. This system can also be utilized as a bridge to surgery by reducing the perioperative risk through improving clearance of infection, reducing the risk of septic pulmonary embolism, and improving hemodynamics. Multidisciplinary care is recommended for all patients of IE for better outcomes.
